# Modelling VA-CNT surface morphology for pollutant adsorption from aqueous media†

**DOI:** 10.1039/d4na00915k

**Published:** 2025-01-22

**Authors:** Inês E. Oliveira, Ricardo M. Silva, Cláudia G. Silva, Rui F. Silva

**Affiliations:** a CICECO – Aveiro Institute of Materials, Department of Materials and Ceramic Engineering, University of Aveiro 3810-193 Aveiro Portugal rsilva@ua.pt; b Laboratory of Separation and Reaction Engineering – Laboratory of Catalysis and Materials (LSRE-LCM), Faculty of Engineering, University of Porto Rua Dr Roberto Frias s/n 4200-465 Porto Portugal; c ALiCE – Associate Laboratory in Chemical Engineering, Faculty of Engineering, University of Porto Rua Dr Roberto Frias 4200-465 Porto Portugal

## Abstract

The absorption of organic dyes from aqueous medium by vertically aligned carbon nanotubes (VA-CNTs) is an ongoing research topic, as these nanostructures present outstanding physical and chemical properties that may favor their interaction with organic pollutants. The growth temperature variation yields different VA-CNT surface morphologies ranging from wave-like patterns to patternless or planar patterns. Here, we demonstrate the effect of the interaction between the intrinsic oxygen concentration of the VA-CNTs grown at different temperatures (650 °C and 700 °C) on the adsorption of cationic (Rhodamine B – RhB) and anionic (Methyl Orange – MO) dyes from aqueous media through UV-vis spectrophotometry studies. The XPS technique was applied to estimate the oxygen percentage (O at%) present on the nanotubes as follows: 0.62% on the wavy patterned VA-CNTs and 2.32% on VA-CNTs without a pattern. The highest adsorption performance was accomplished by VA-CNTs without a pattern for RhB dye, attributed to electrostatic interactions, where the positively charged RhB is attracted to the negatively charged nanotube surface. In this way, VA-CNTs have shown adsorption characteristics crucial for wastewater decontamination.

## Abstract 2

Catalyst engineering of vertically aligned carbon nanotubes (VA-CNTs) through their spontaneous wave-like growth and intrinsic oxygen concentration.

## Graphical abstract

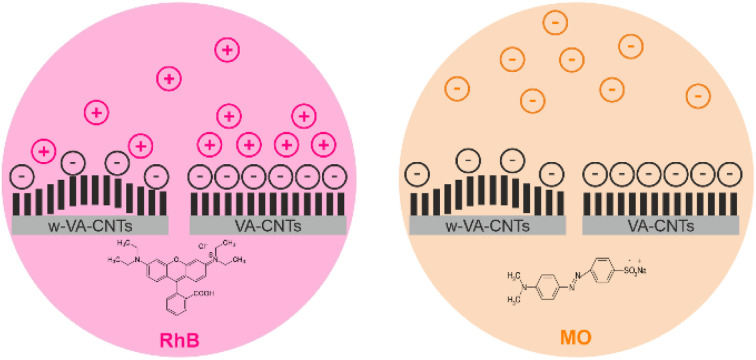

## Introduction

Adsorption is a highly effective method commonly used for organic pollutant removal from industrial wastewater streams. Organic dyes used in the textile and cosmetic sectors pose a significant environmental threat due to their toxicity and resistance to degradation. Dye molecules in the textile industry can be classified based on their chemical structure, which influences their colour and properties. These dyes are widely classified into two main groups: cationic and anionic dyes, each with distinct chemical structures. Cationic dyes, such as Rhodamine B (RhB) and Methylene Blue (MB), are characterized by the presence of cationic functional groups. On the other hand, anionic dyes, including Methyl Orange (MO) and Phenol Red (PR), are negatively charged and contain anionic functional groups (sulfonic or carboxylic acid groups).^1^

The adsorption processes are versatile, capable of removing various dye molecules, and are a simple, economical solution that can be easily scaled for wastewater treatment. A wide range of materials, including alumina, activated carbon, clays, silica gel, composites, zeolites, biomass, and both biological and polymeric substances, have been applied as adsorbents for the removal of contaminants from environmental water.^2^ Recent advances in new materials, such as carbon nanomaterials, have improved adsorption efficiency, and water purification in different industries, even at trace contaminant levels.

Carbon-based materials (CBMs) are highly effective in removing organic pollutants from water due to their large surface area, high porosity, low density, and strong affinity for contaminants. The study of these materials has expanded rapidly, driven by their unique properties and potential applications in chemistry, physics and engineering. Common carbon-based materials include activated carbon, graphene, carbon nanotubes, carbon nanofibers, biochar, and carbon aerogels, which rely on large surface areas for effective adsorption through physical and chemical interactions.^2–4^

**Fig. 1:**
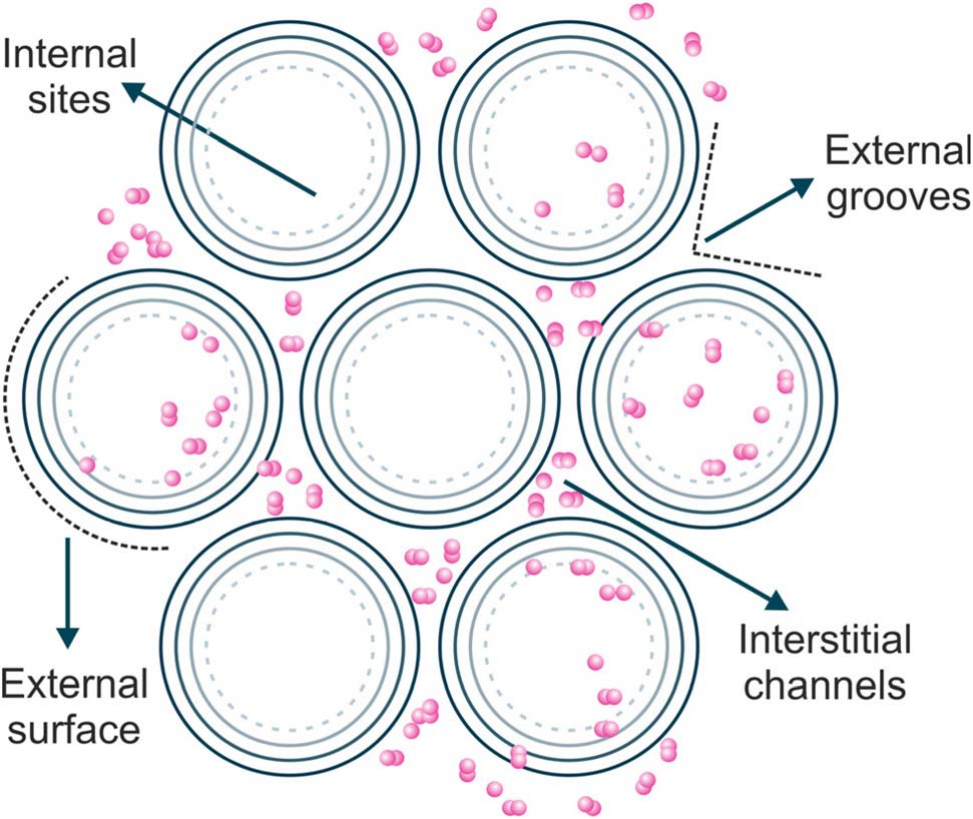
Schematic representation of possible adsorption sites for the interaction of contaminants with carbon nanotubes, adapted from Ref. 8.

Activated carbon (AC) presents several forms with unique surface properties, including surface area, pore size, pore volume, and functional groups, which can be modified based on the precursor, activating agent, activation temperature, and production method. This carbon material is known for its large surface area (500–3000 m^2^ g^−1^), low cost, and high porosity. AC is widely used for water treatment, though its high cost and limited regeneration capabilities can sometimes limit its use.^2,4,5^ The excellent adsorption abilities of carbon nanotubes (CNTs) for organic contaminants are mainly due to their unique structural and functional characteristics. CNTs have remarkable strength and unique electrical, mechanical, and thermal properties. Their high surface area, tuneable surface chemistry, and surface oxygen-containing functional groups enhance their adsorption performance.^4,6^ On the other hand, CNTs are different from ACs because their structure on the atomic scale is more well-defined and uniform. In adsorption to ACs, pore diameter distribution and adsorption energy distribution are relevant parameters, which is not the case for CNTs. Considering that, with CNTs, one can directly access several well-defined adsorption sites available for adsorbed molecules.^7^ There are four possible adsorption sites, on the CNT surface, for organic pollutants (Fig. 1): (i) the internal hollow space inside nanotubes, (ii) the external surface of the outermost nanotubes, (iii) interstitial channels between individual nanotubes, and (iv) external grooves along the outer surface of nanotube bundles. In multi-walled CNTs (MWCNTs), there is an additional adsorption space available between the walls. The adsorption efficiency depends on factors such as CNT purity, structural characteristics (surface area, porosity, and charge density), and surface functional groups.^3,5–7^

The purpose of this work is to study the influence of the intrinsic oxygen concentration of the VA-CNTs on the sorption of organic contaminants (*e.g.*, dyes) from aqueous media. The VA-CNTs are expected to function as an efficient adsorbent with increased adsorption capacity, selectivity, and a short response time. The presence of oxygen may alter the adsorption of organic contaminants on the VA-CNT surface. Moreover, a general relationship between the oxygen concentration and the chemical nature of the organic dyes was established by means of adsorption experiments. In this context, the VA-CNT growth approach was based on reducing the activation energy of the TCVD reactions with good control over the growth parameters. TCVD can grow VA-CNTs on predefined areas on a substrate previously coated with a metal (Ni) catalyst. VA-CNT growth requires the formation of active nanosized metal catalyst particles by a reduction step (H_2_ atmosphere), where the growth of VA-CNTs occurs. Its catalytic activity depends on the diffusion and solubility of the carbon within the nanosized catalyst particles. Normally, an oxide buffer layer such as SiO_2_ and Al_2_O_3_ is often employed with the metal catalyst to improve the catalyst's performance and consequently the VA-CNT yield. As a result, high surface-to-volume ratio nanostructures are obtained.

## Experimental

The process of producing wave-like vertically aligned carbon nanotube arrays (w-VA-CNTs) comprises several steps. First, Si/SiO_2_ substrates are coated with a buffer layer followed by a metallic Ni catalyst thin film by physical vapor deposition (PVD). Then, the thermal annealing of the thin film and, finally, the growth of carbon nanotubes were performed *via* thermal chemical vapor deposition (TCVD).

### PVD multilayer catalyst preparation

Polished Si wafers with thermally grown SiO_2_ (200 nm) and a single side polished Si wafer (Siegert Wafer, 〈100〉 orientation) were used as substrates. The multilayer catalyst preparation with Al_2_O_3_ (35 nm) or TiO_2_ (40 nm) or Cr_2_O_3_ (40 nm) and the Ni thin film was accomplished by means of PVD, in particular DC magnetron sputtering. The DC power is applied to the target foil (Al, Ti or Cr and Ni) in an inert or reactive atmosphere of argon (Ar; 10 sccm) or a mixture of argon and oxygen (Ar/O_2_; 10/4 sccm), with a working pressure of 5.0 × 10^−4^ mbar. Afterwards, bare Si, Si/SiO_2_ substrates and sputtered Si/SiO_2_/Al_2_O_3_, Si/SiO_2_/TiO_2_, and Si/SiO_2_/Cr_2_O_3_ substrates were coated with a DC magnetron sputtered Ni thin film of 12 nm thickness. A preliminary set of experiments involving the use of different thicknesses of Ni thin films were undertaken in order to understand the deposition rate of Ni. As the VA-CNT array growth is highly related to the metallic nano-sized catalyst particles, especially in catalytic TCVD, resulting from the restructuring of the Ni uniform thin film. Table S1† summarizes the experimental parameters corresponding to the multilayer catalyst preparation.

### TCVD synthesis of VA-CNT arrays

**Fig. 2:**
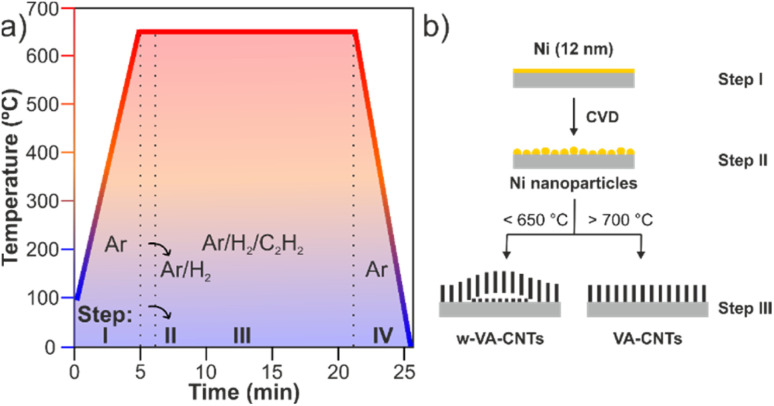
Schematic illustration of the different process steps for growing VA-CNTs by TCVD (a). Schematic representation of growth steps of w-VA-CNT and VA-CNT arrays (b) (drawing not to scale).

The synthesis of w-VA-CNT and VA-CNT arrays was performed in a thermal chemical vapor deposition reactor. The growth of nanotubes can be divided into four steps (Fig. 2a): heating (I); annealing (II) to expose the film to nanoparticles; growth of the nanotubes (III); and cooling (IV). The first and last steps aim to heat and cool the samples in an argon atmosphere. The pretreatment (step II) is conducted in a reducing atmosphere of H_2_/Ar (500/200 sccm) for 1 min. The samples were grown (step III) on the as-prepared substrates, for 15 min, using a gas mixture of C_2_H_2_/H_2_/Ar (10/100/400 sccm). The total gas flow was fixed at 510 sccm, for all the experiments. Initially, several depositions were carried out by varying the temperature from 450 to 750 °C until the desired temperature was established at 650 °C. Table S2† summarizes the experimental parameters corresponding to the several VA-CNT syntheses carried out, where different values of temperatures were tested.

The gas mixture ratio was varied throughout the growth process on Si/SiO_2_ substrates. This experiment consists of understanding the influence of H_2_ inside the chamber and, to this end, its quantity was varied throughout the steps. In other words, several depositions were made, at 650 °C, where the sample heated for 6 min (step I) and, in all depositions, the pre-treatment was suppressed (step II). The growth of nanotubes (step III) consists of the following ratios of C_2_H2/H_2_/Ar gases: 10/0/500, 10/50/450 and 10/100/400 sccm, where the amount of carbon source remained constant, H_2_ was varied, and the total volume was adjusted by the amount of Ar.

**Fig. 3:**
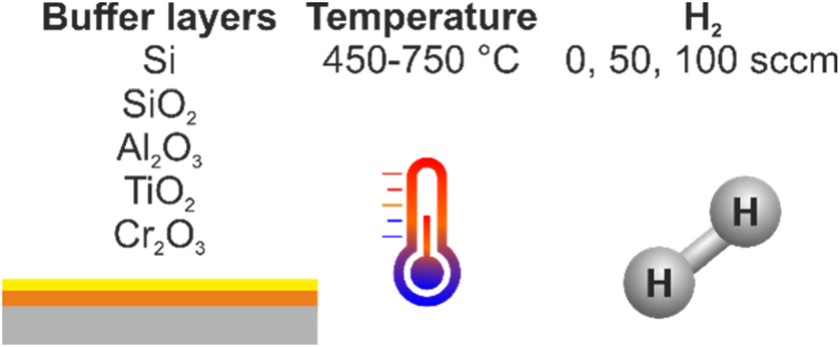
Schematic representation of the several parameters studied during this work.

Therefore, to study and understand the formation of wave patterns present on the nanotubes and the growth mechanism, several parameters were studied: buffer layers, synthesis temperature and amount of hydrogen inside the chamber (Fig. 3).

### Materials characterization

The steps of the experimental procedure were well characterized in an attempt to understand the phenomenon of waves in nanotube forests. In order to determine the nanoparticle diameter and its distribution over the substrate, the height and morphology of the forests, the samples were characterized by scanning electron microscopy (SEM) (Hitachi SU70), performed at 15 kV. Also, the substrate was analysed to determine the diameter and the distribution of the Ni nanoparticles after the growth of VA-CNTs. This procedure was carried out by submerging the forests in acetone and using an ultrasonic bath.

The internal structure of the nanotube was investigated by high resolution transmission electron microscopy (HRTEM) (JEOL JEM-2200FS), performed at 200 kV. The samples for HRTEM measurements were prepared by dry adhesion of the CNTs to a holey carbon film supported on a copper grid.

The degree of crystallinity of the carbon nanotubes and the CNT structure after the experiments was determined by Raman spectroscopy (Horiba Jobin Yvon HR800 spectrometer), under a 532 nm laser line. X-ray diffraction (XRD) measurements were performed on a PANalytical X'Pert PRO diffractometer using Cu Kα radiation (*λ* = 1.54060 Å). The operating conditions were set to a *θ*–2*θ*° range of 8–80° and a step size of 0.02°.

Wettability measurements were used to measure the contact angle, at room temperature, using a drop of deionized water (H_2_O, Milli-Q, drop volume 3 mL) in a commercial system (Dataphysics OCA).

X-ray photoelectron spectroscopy (XPS) measurements were performed in an ultrahigh-vacuum system with a 2 × 10^−10^ mbar base. The XPS equipment is fitted with a hemispherical electron energy analyser (SPECS Phoibos 150), a delay-line detector, and a monochromatic AlKα (1486.74 eV) X-ray source. High-resolution spectra were recorded at a normal emission take-off angle with a pass-energy of 20 eV, affording a 0.5 eV overall instrumental peak broadening. The binding energy of each element was calibrated using the C 1s peak (284.6 eV). For oxygen percentage (O at%) determination, the XPS core-level spectra of O 1s were deconvoluted using XPSPeak4.1 software using Gaussian–Lorentzian (G–L) fitting functions after Shirley-type background subtraction.

### Adsorption experiments

The adsorption experiments were performed using Rhodamine B (RhB) and Methyl Orange (MO) as adsorbates and all the carbon nanotubes as adsorbents. RhB and MO were chosen according to the chemical structure: cationic and anionic dyes, respectively. These dyes were chosen to study the influence of the surface chemistry of w-VA-CNT arrays during the adsorption experiments. The experiments were conducted in a cylindrical glass reactor filled with 20 mL of a 5 mg per L dye aqueous solution. The solution was continuously purged with an airflow. The solution was kept in the dark for 240 min and at certain times, samples were collected to assess the concentration variation of the dye by UV-vis absorption spectroscopy measurements, using ultraviolet-visible spectrophotometry (UV-vis) (Shimadzu LISR-3100 UV-vis-NIR). The pH of the solution was measured before and after each experiment. The initial and final values were 5.3. The samples have a geometrical area of 1 cm × 1 cm. The recyclability of w-VA-CNTs was assessed by repeating three consecutive adsorption reaction tests using a fresh dye solution for each cycle. Specifically, after completing each degradation cycle, the sample was immersed in deionized water for a few minutes, followed by drying in a furnace at 60 °C overnight.

## Results and discussion

### Ni catalyst–support interactions

One of the most key factors that affect CNT nucleation and growth, and particularly the successful growth of vertically aligned CNT structures, is the catalyst–substrate interaction. Therefore, several parameters that can affect the interactions between the catalyst and the substrate were studied, including the thickness of the catalyst film, the annealing time, and the amount of hydrogen (H_2_) inside the chamber.

**Fig. 4:**
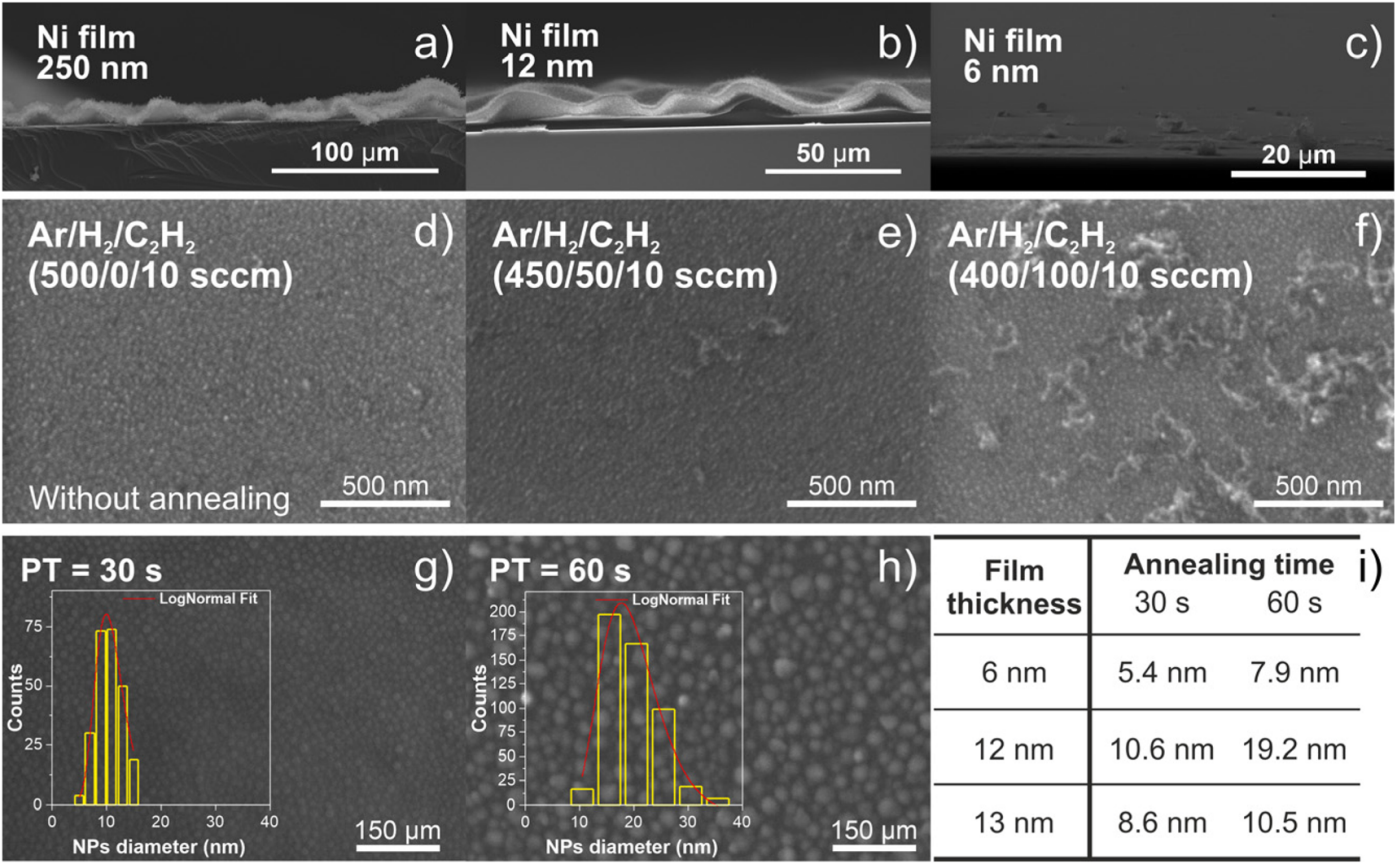
Tilted side view SEM image of carbon nanotube growth, at 650 °C, on a substrate with 250 nm (a), 12 nm (b) and 6 nm (c) Ni films. Top-view SEM image of the Si/SiO_2_ substrate, with the 12 nm Ni film, submitted to synthesis with no annealing step and varying the amount of H_2_ inside the chamber, during the growth step, at 650 °C with 0 sccm (d), 50 sccm (e) and 100 sccm (f) of H_2_. Top view SEM images of Ni nanoparticles grown, at 650 °C, on the 12 nm Ni film for 30 s (g) and 60 s (h) of annealing. The table in (i) summarizes the Ni nanoparticle diameter range, formed after annealing, for different thickness films.

The prepared substrates with Ni thin films were used to grow the carbon nanotubes, which depends on the formation of nanoparticles from a metal catalyst. The metallic catalyst is one of the major factors in nanotube synthesis and Ni is known to be active as a catalyst in the synthesis of CNTs. However, it is necessary to consider its thickness value. The variation of the deposition time allows the deposition of Ni films with different values of thickness and therefore the assessment of the deposition rate. There is a linear increase of film thickness with the deposition time (not shown), as expected for the intrinsic characteristics of the magnetron sputtering deposition technique.^9^ The deposition rate of the Ni films was 0.994 nm min^−1^ determined by linear fitting, which means that the film thickness can be adjusted easily using the deposition time. Moreover, these data also suggest good control of the film thickness at higher values (around 250 nm) or at lower values (around 12 nm) (Fig. 4a–c).

The information on the nucleation and the growth process of CNTs during the synthesis is of high significance. In fact, nanotube–nanotube and nanotube–support interactions and the order and activity of the Ni catalytic sites have a significant effect on the final configurations (isolated, tangled, aligned or wavy configuration) of the grown CNTs. The use of H_2_ gas improves the performance of the Ni catalyst nanoparticles towards the CNT growth. Fig. 4d–f highlight the importance of the pretreatment step (annealing) in a reducing atmosphere (Ar/H_2_), before the CNT growth step, and the H_2_ concentration variation during the CNT growth step (Ar/H_2_/C_2_H_2_). There is almost no growth of CNTs without H_2_ or low H_2_ concentration present during the deposition process, which indicates that a suitable H_2_ concentration can make the growth of CNTs more efficient. Therefore, it is crucial to maintain a balance between carbon and hydrogen radicals in the reaction system to ensure proper CNT growth. Based on the above observation and analysis, the contribution of H_2_ in the pretreatment step is crucial for Ni nanoparticle activation, maximizing their catalytic activity.

As mentioned above, the Ni films were used as catalysts to grow carbon nanotubes, and as reported in the literature, the thickness of the metallic catalyst film influences the CNT growth by TCVD.^10,11^ In this line, three samples with lower values of thickness were used. After the annealing process (as schematically represented in step II of Fig. 2), the continuous film of Ni is broken down into catalyst nanoparticles with a narrow distribution size, where the CNT growth takes place. For this reason, the catalyst nanoparticles were characterized in terms of size distribution as a function of the annealing time duration (30 or 60 seconds), produced at 650 °C (Fig. 4g–i). For comparison purposes, three different Ni films with the thicknesses of 6, 12 and 13 nm were used to understand the effect of the annealing time duration in the film surface morphology. In the case of the 12 nm thick film, the surface morphology with two different annealing times can be observed, as shown in Fig. 4g and h. As the annealing time increases, the Ni catalyst nanoparticle diameter size also increases, and they appear as a particle-like film.

The table in Fig. 4i summarizes these diameter sizes as a function of the annealing time duration and film thickness. Generally, the catalyst nanoparticle diameters are directly related to the final diameter of the carbon nanotubes. Moreover, these results suggest that the Ni catalyst film can be tuned to produce the desirable catalyst nanoparticle size distribution regarding the application of the carbon nanotubes.

After the formation of Ni nano-sized particles, during the annealing process (60 s) (step II), substrates with different thicknesses (6, 12 and 250 nm) underwent the nanotube growth stage (step III). The SEM images in Fig. 4a–c show wave-like carbon nanotube (w-VA-CNT) array formation from the thicker catalyst film. However, no growth was found in the thinner catalyst film (6 nm). The fact that there is no growth with the 6 nm thick Ni film may be due to the smaller diameter of the nano-sized particles. Therefore, the thickness of the Ni thin film was fixed at 12 nm and an annealing time of 60 s (1 min) for all the samples was used during the work.

### Effects of the supporting layer on the morphologies of Ni catalyst nanoparticles and subsequent growth of CNTs by TCVD

The structure of the forest depends on the sample preparation and synthesis conditions. Having a straightforward approach to tailoring the structure of the forests would be advantageous, so that it is both reproducible and easily controllable. One powerful approach to controlling the structure of the forest is to engineer the catalyst by controlling the thickness of the catalytic thin metal film. A thin metal film is widely used as a standard catalyst for CNT forest growth. When this film is annealed, it converts into well-isolated, individual nanoparticles that function as catalysts for nanotube growth.

**Fig. 5:**
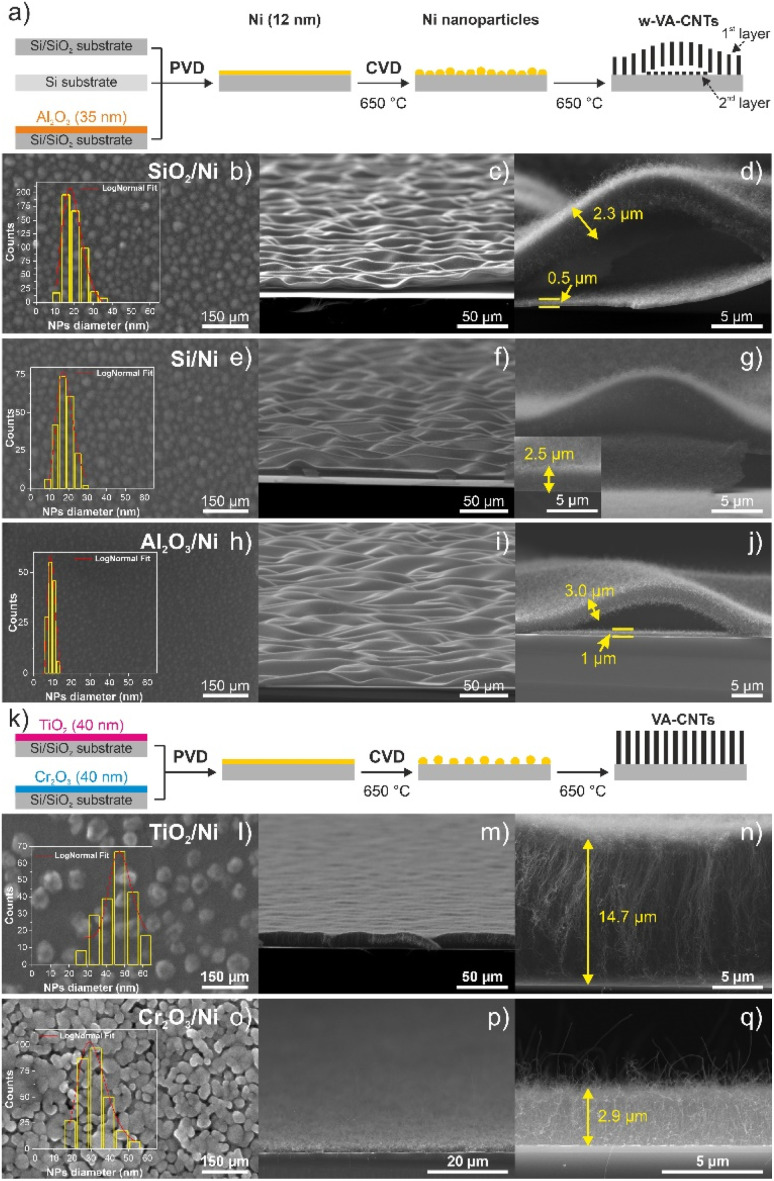
Schematic representation of growth steps of w-VA-CNTs (a) and VA-CNTs (k) on different substrates and buffer layers: SiO_2_, Si substrate, Al_2_O_3_, TiO_2_ and Cr_2_O_3_ thin films. Top-view SEM image of the Ni nano-sized catalyst particles at 650 °C for 1 min of annealing pretreatment (b, e, h, l and o – left column), tilted (c, f, i, m and p – middle column) and cross-sectional SEM images (d, g, j, n and q – right column) of the w-VA-CNTs and VA-CNTs grown at 650 °C for 15 min on SiO_2_/Ni, Si/Ni, Al_2_O_3_/Ni, TiO_2_/Ni and Cr_2_O_3_/Ni, respectively. Insets show the typical particle size distribution histogram; red line is the log-norm fit of the bin center points.

The catalyst nanoparticle morphology can be changed with an intermediate buffer layer between the substrate and the catalyst film. These layers are used to prevent the diffusion of the catalyst into the substrates. The influence of buffer layers on the formation of Ni catalyst particles and the VA-CNT growth with a wave or planar pattern was studied under the same conditions of TCVD deposition, at 650 °C with a growth time of 15 min. Ni nano-sized catalyst particles were grown using five different layers: Si (bare), SiO_2_ (from the substrate), Al_2_O_3_, TiO_2_ and Cr_2_O_3_ thin films. To further understand the growth of wave-like or planar VA-CNTs, the nanotubes and catalyst nanoparticles with the different buffer layers were characterized by SEM (Fig. 5).

Based on Fig. 5, the formation of two types of nanotube morphology can be observed using different types of buffer layers: (i) Si, SiO_2_ and Al_2_O_3_ – wave-like vertically aligned carbon nanotubes (w-VA-CNTs) and (ii) TiO_2_ and Cr_2_O_3_ – planar vertically aligned carbon nanotubes (VA-CNTs). The most visible difference between the two types of morphologies under study is the size and distribution of the Ni nanoparticles that formed during the annealing step, conducted under the same conditions. The Ni nano-sized particles formed on SiO_2_, Si and Al_2_O_3_ are homogeneously distributed over the substrate with a high density of nanoparticles, in the range of 6.5 × 10^10^–1.5 × 10^11^ cm^−2^, with an average diameter of 19.22, 18.31 and 9.56 nm, respectively. Concerning the nanoparticles formed on TiO_2_ and Cr_2_O_3_ are more dispersed over the substrate (1.4 × 10^10^ and 4.2 × 10^10^ cm^−2^), and its diameter is higher and around 47.84 and 31.03 nm, correspondingly. This means that Ni nanoparticles have higher mobility and low adhesion on TiO_2_, compared to SiO_2_. This favours coalescence during pretreatment or growth and the formation of less-dense, larger nanoparticles and consequently an increase in the nanoparticle inter-distance. As a result, CNTs are grown from different-sized Ni nanoparticles at different rates.

SiO_2_/Ni, Si/Ni and Al_2_O_3_/Ni appear to show the highest stability and the lowest ripening rate, while particle growth *via* coalescence and/or Ostwald ripening is apparent for TiO_2_/Ni and Cr_2_O_3_/Ni, where large catalyst particles appear on TiO_2_/Ni. This finding reflects the high surface energy of Ni compared to that of TiO_2_ and the self-diffusivity of Ni due to low adhesion energy to the TiO_2_ support.^12,13^

**Fig. 6:**
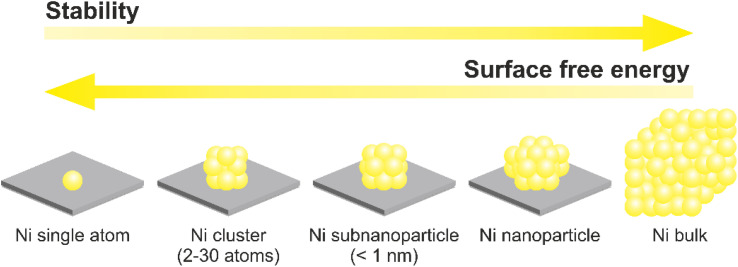
Schematic illustration of the transition from Ni bulk to the supported Ni single atom, adapted from Ref. 14.

The Ostwald ripening behaviour of the catalyst depends strongly on the support; the Ostwald ripening rate appears to be highest with TiO_2_/Ni, intermediate with SiO_2_/Ni, Si/Ni and Cr_2_O_3_/Ni and the least with Al_2_O_3_/Ni. Considering that the highest surface energy is assigned to a single Ni atom, it needs to be strongly stabilized on the support to prevent its migration for the purpose of binding, in order to reduce the free surface energy (Fig. 6). A summary of the physical properties of the catalysts supported on the different substrates is presented in Fig. 6.

**Fig. 7:**
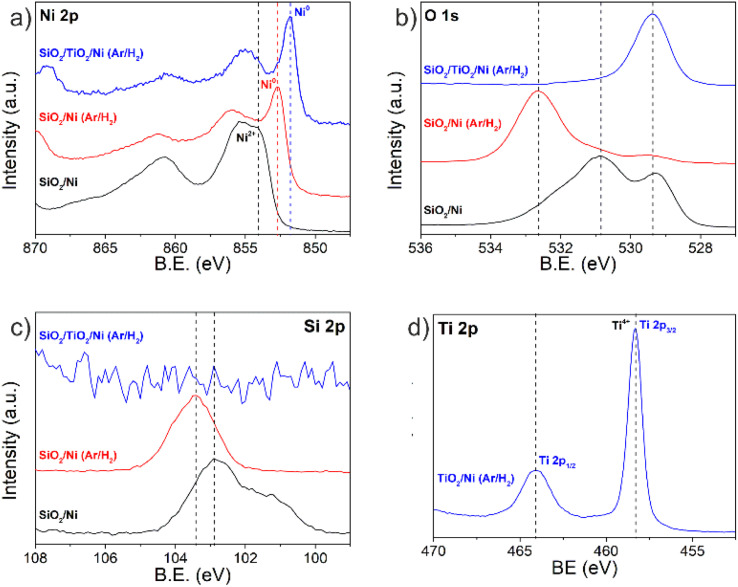
XPS core level spectra of (a) Ni 2p, (b) O 1s, (c) Si 2p and (d) Ti 2p, after the pretreatment step in a reducing atmosphere (Ar/H_2_).

It can be clearly seen in Fig. 5 that the CNT vertical growth with a wavy-like morphology depends on the Ni catalyst–support interaction. To understand this, we performed XPS analysis of the Ni nanoparticles before the growth step, on SiO_2_ and TiO_2_ buffer layers, after the pretreatment step in a reducing atmosphere (Ar/H_2_). XPS can be used to determine the electronic state of Ni. The main binding energies for Ni are in various oxidation states (from Ni^0^ to Ni^3+^), as shown in Fig. 7a and Table 2. Considering that the Ni was deposited on a thermally oxidized Si substrate (Si/SiO_2_), several Ni phases (metallic and Ni oxide) were observed in the Ni 2p spectra (Fig. 7a). In other words, the deposition of Ni results in a metallic layer that is further oxidized through manipulation in air. The oxidation state of Ni changes after the pretreatment step, and the Ni 2p on the TiO_2_ support layer shifted toward lower binding energy as compared to Ni 2p on SiO_2_, indicating that the Ni is reduced in both cases; however, the Ni^0^ on TiO_2_ seems to be present in a greater quantity. In fact, larger Ni^0^ nanoparticles with a high catalytic activity were observed on the TiO_2_ support (*i.e.*, buffer) layer in comparison to the SiO_2_ support layer. The use of TiO_2_ was particularly effective in suppressing the wavy-like morphology yielding planar VA-CNTs. The TiO_2_ support layer is extremely stable and is not expected to be reduced under standard growth conditions and the Ti did not undergo any chemical evolution and remained as Ti^4+^, as indicated by the characteristic Ti 2p peaks (Fig. 7d). Additionally, the Ti electronegativity is the lowest (1.5 on the Pauling scale), resulting in the largest charge transfer from the Ti to the O atoms, and, in consequence, the lowest binding energy of the related O 1s peak (Fig. 7b). It is reasonable to state that the catalyst is active in its metallic state only, and if oxidized, it requires activation, *i.e.*, at least partial surface reduction prior to growth. The highest intensity Si 2p peak, located at 103.4 eV, can be associated with photoelectrons emitted from Si atoms in SiO_2_ (Fig. 7c). It can be suggested that, during the pretreatment step, the Ni does not react with the SiO_2_ overlayer of the Si substrate. On the other hand, the binding energy around 102.9 eV can be assigned to Si 2p (SiO_2_/Ni as-deposited), suggesting the formation of a non-stoichiometric silicon suboxide.^15^

The carbon nanotubes grown from these Ni catalyst particles are shown in the SEM images in Fig. 5 – middle and right columns. From these images, it is possible to observe that when using Si, SiO_2_ and Al_2_O_3_, as buffer layers, w-VA-CNTs are obtained with a wave pattern in the range of 2.3–3.0 μm in height and these forests have detached from the substrate (*i.e.*, lift-off). Conversely, to obtain planar VA-CNTs, TiO_2_ and Cr_2_O_3_ as buffer layers are used and their height is around 14.7 μm, when grown on TiO_2_/Ni and 2.9 μm for Cr_2_O_3_/Ni. Furthermore, the Ni catalyst film can be tuned to produce the desirable catalyst nanoparticle size distribution regarding the application of the carbon nanotubes.

**Fig. 8:**
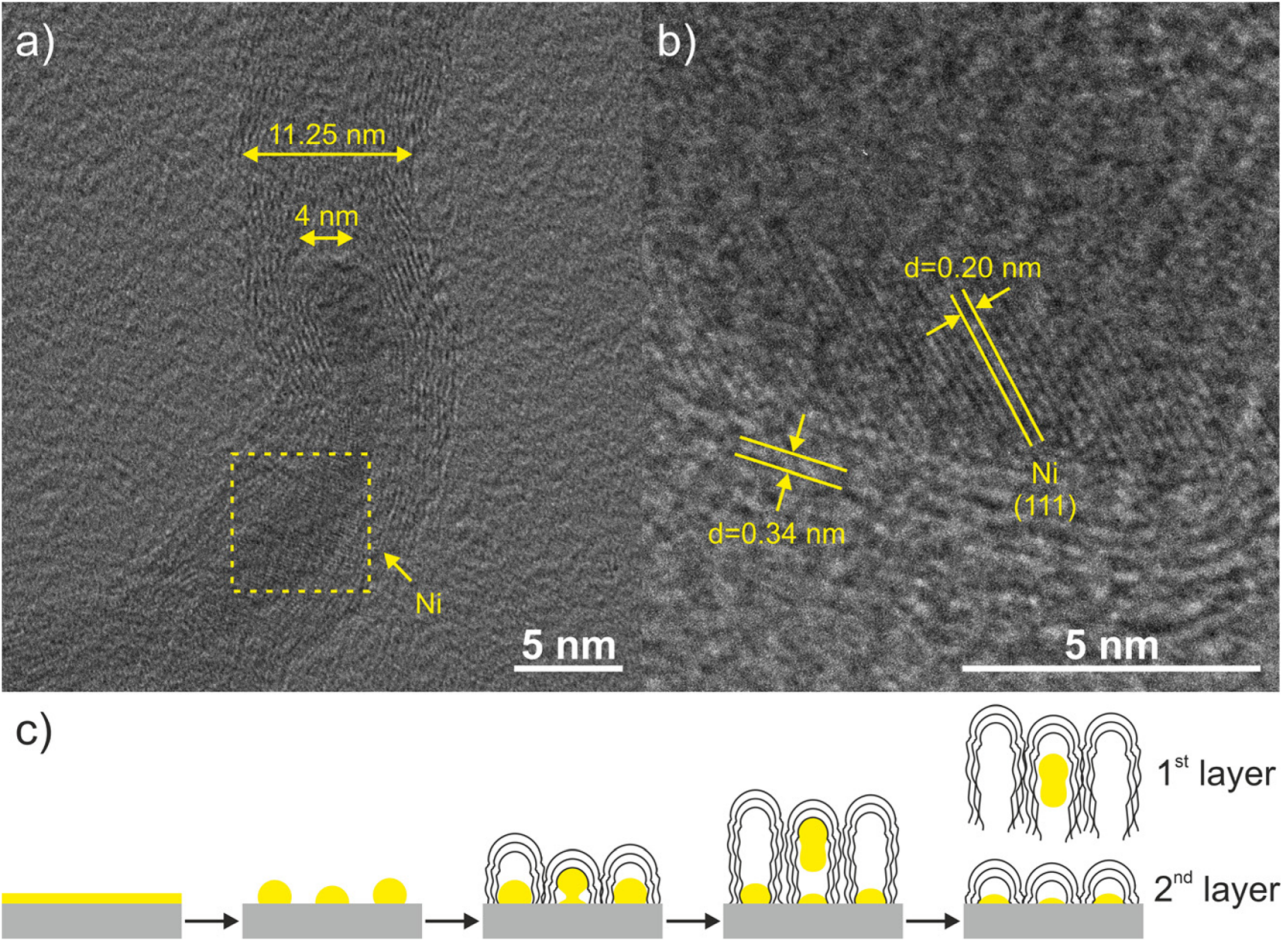
HRTEM images of nanotubes grown on SiO_2_/Ni (a) and taken from the area depicted by the yellow square (b). Schematic representation of nanotube growth of the 1st and 2nd layers (c).

Understanding the interface structure between the Ni catalyst and the grown CNT is key to unravelling the subsequent growth mechanism. From our observations, the wave-like pattern can be seen as the formation of a three-dimensional network, which tends to corrugate during CNT growth. The area of the CNT network increases accordingly and mismatches that of the supporting area. As a consequence, the mismatched area causes the network lift-off and loss of contact with the SiO_2_ support.^16,17^ This finding could be explained by the thermal motion that eventually results in detachment of the lengthening CNT. After each detachment, the next CNT nucleates and grows after carbon supersaturation on the catalyst particle is re-established and capable of nucleating a second layer of VA-CNTs, as shown in Fig. 5d and schematically illustrated in Fig. 8c. This also suggests that other complex features such as catalyst–support interactions driven by interfacial strain or chemical energies may play a key role in the nucleation of the CNTs and further formation of the wave-like pattern. It is important to point out that the observations may be specific to the deposited Ni catalyst and the TCVD deposition process (*e.g.*, gas mixture Ar/H_2_/C_2_H_2_) performed in this study and that if other catalysts or gas mixtures are used the outcome may be different.

**Fig. 9:**
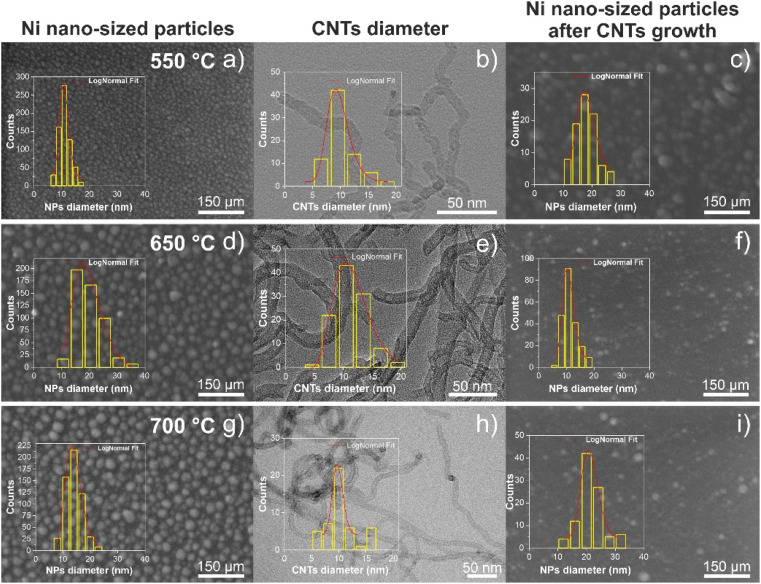
Top-view SEM image of the Ni nano-sized catalyst particles, for 1 min of annealing pretreatment, at 550 °C (a), 650 °C (d) and 700 °C (g). Overview BF-TEM image of CNTs grown at 550 °C (b), 650 °C (e) and 700 °C (h). Top-view SEM image of the Ni nano-sized catalyst particles after the removal of the w-VA-CNTs and VA-CNTs, grown at 550 °C (c), 650 °C (f) and 700 °C(i). Insets show the typical particle size or CNT diameter distribution histograms, and red line is the log-norm fit of the bin center points.

The HRTEM image (Fig. 8a) shows multi-walled CNTs (8–12 walls) with defective walls, presenting a characteristic interlayer spacing of 0.34 nm, which can be assigned to the (002) plane of graphite. In some cases, we detected Ni nanoparticles inside of the nanotubes, as shown in Fig. 8b. The observation of a lattice spacing of 0.20 nm corresponding to the metallic Ni (111) crystallographic plane suggests that the catalytic nano-sized particles are in a molten liquid state at the deposition temperature and the stretching force causes them to elongate and finally break. This observation is supported by the results depicted in Fig. 9.

The Ni nano-sized particles were characterized before and after the growth step; to this end, the CNTs were removed from the Si/SiO_2_ substrate. The nanotubes under study at this stage were all grown at 650 °C, which corresponds to the middle row of Fig. 9d–f. As shown at the beginning of this section, the Ni nano-sized particles are distributed homogeneously throughout the substrate. The internal structure of CNTs was investigated using BF-TEM images, as well as the internal and external diameters. The inner diameters of various w-VA-CNTs, grown at 650 °C, were determined from high-resolution TEM images, as shown in Fig. 8. The inner diameter of the tube was found to be 4 nm. Most of the w-VA-CNTs have an external diameter in the range of 5.91–19.69 nm, and the average diameter was found to be 11.25 nm. The external diameters were measured from several overview images, creating a particle size distribution histogram (shown in the inset). The size of Ni catalyst nano-sized particles is related to the diameter of the nanotubes, which is confirmed because the diameter of the nanotube is in the range of the Ni catalyst nanoparticle diameter. These values are in agreement with the diameter of the Ni nano-sized particles measured on the substrate after removing the nanotubes (10.74 nm), as shown in Fig. 8f.

Only three types were further characterized: w-VA-CNTs grown on SiO_2_/Ni and Al_2_O_3_/Ni and VA-CNTs grown with TiO_2_/Ni. Raman spectroscopy was used to evaluate the degree of crystallinity of the prepared carbon nanotube arrays. There are two Raman characteristic peaks for CNTs. The D-band (∼1350 cm^−1^) represents the structural defects and the formation of amorphous carbon on the nanotube walls. The G-band (∼1580 cm^−1^) arises from the E_2g_ vibration of the graphite plane, characterizing the ordered graphitization of the CNTs. The ratio between the integrated areas of D and G bands (*I*_D_/*I*_G_) is used to evaluate the extension of these structural defects; *i.e.*, lower ratio values indicate a good degree of crystallinity of the CNTs.

In this case, the ratio *I*_D_/*I*_G_ of the w-VA-CNT arrays grown on SiO_2_/Ni was estimated to be around 1.45, pointing to a higher degree of disorder or defects compared with CNTs with a good degree of crystallinity.^18,19^ The *I*_D_/*I*_G_ ratios of the CNTs with Al_2_O_3_ and TiO_2_ are almost the same, despite the difference in their morphology. The first one has a wave-like pattern and the other one is vertically aligned to the substrate. The same trend is observed with the water contact angle presented in Table 1. The water contact angle (*θ*_c_) measurements obtained for the CNT arrays produced with different buffer layers do not seem to correlate with the different morphologies.

**Table 1:** Dependence of the growth rate on the Ni nano-sized catalyst particle diameter (Ni thin film of 12 nm thickness). Intensity ratios of integrated areas of D and G bands and water contact angles (*θ*_c_) of the different CNT arrays obtained. Synthesis period of 15 min at 650 °C

Buffer layer type	Ni nano-sized particle diameter (nm)	Ni nano-sized particle density (cm^−2^)	VA-CNT length (μm)	VA-CNT diameter (nm)	*I*_D_/*I*_G_	*θ*c (°)
Si (bare)	18.31	6.5 × 10^10^	2.5	—	—	—
SiO_2_	19.22	8.7 × 10^10^	2.3	11.25	1.45	59.24
Al_2_O_3_	9.56	1.5 × 10^11^	3.0	9.87	1.08	138.59
TiO_2_	47.84	1.4 × 10^10^	14.7	14.20	1.12	149.61
Cr_2_O_3_	31.03	4.2 × 10^10^	2.9	—	—	—

**Table 2:** Species detected by means of XPS measurements after the reducing pretreatment step (Ar/H_2_) and corresponding binding energies (Fig. 7)

Species	Ni 2p_3/2_	O 1s	Si 2p	Ti 2p_3/2_/Ti 2p_1/2_
SiO_2_/TiO_2_/Ni (Ar/H_2_)	851.8 eV	529.4 eV	—	458.3/464.1 eV
SiO_2_/Ni (Ar/H_2_)	852.7 eV	532.6 eV	103.4 eV	—
SiO_2_/Ni (as-deposited)	854.1 eV	529.3 eV	102.9 eV	—
		530.9 eV		

### Influence of synthesis temperature on the morphology of CNTs

**Fig. 10:**
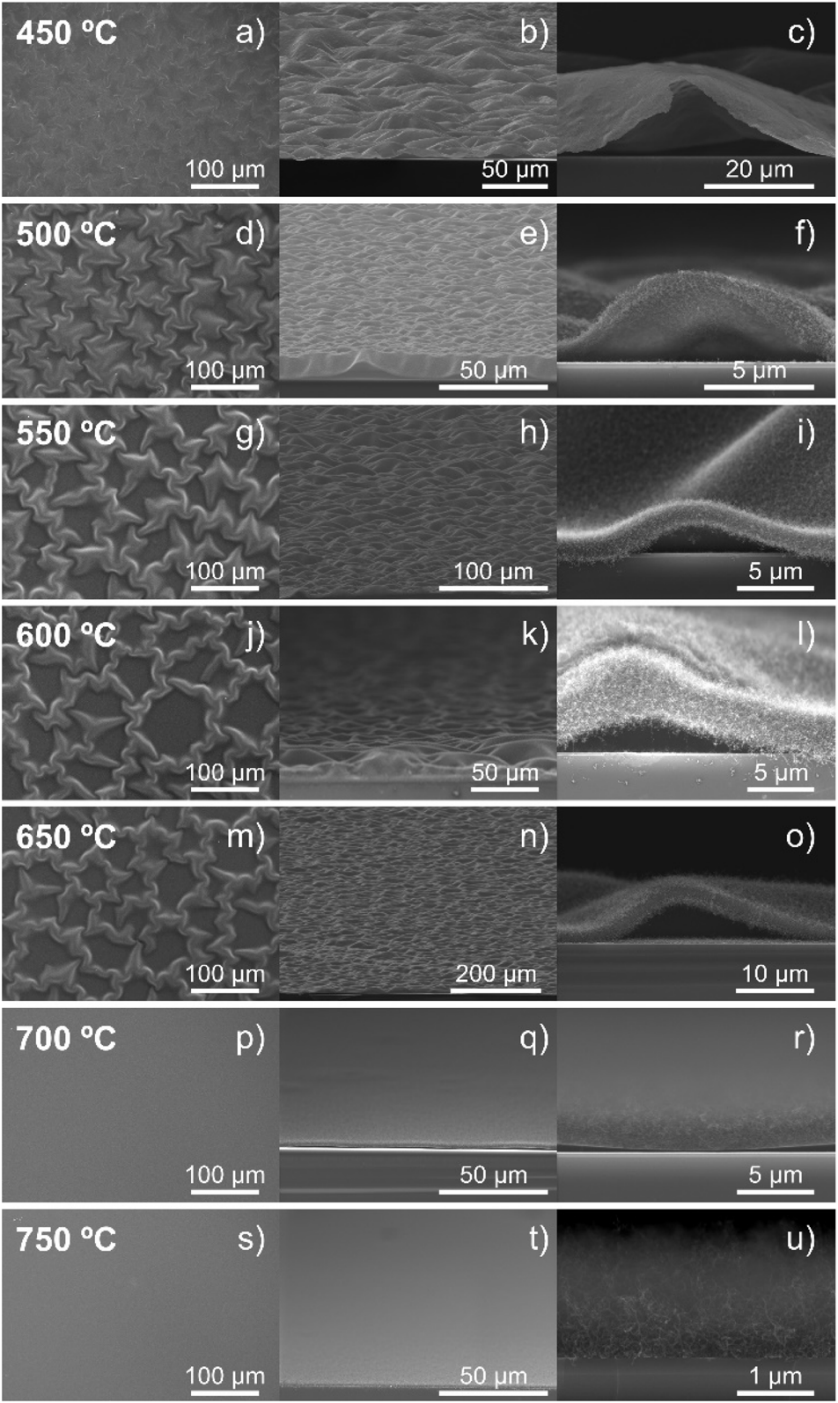
Top (left column), tilted (middle column) and cross-sectional (right column) SEM images of the evolution of w-VA-CNTs and VA-CNTs evolution as a function of the synthesis temperature: 450 °C (a–c), 500 °C (d–f), 550 °C (g–i), 600 °C (j–l), 650 °C (m–o), 700 °C (p–r), and 750 °C (s–u).

The synthesis temperature was critical to the growth of w-VA-CNT arrays. Fig. 10 shows a collection of SEM images of VA-CNTs grown on Si/SiO_2_ at different temperatures ranging from 450 to 750 °C, for 15 min. The main purpose was to investigate the impact of the synthesis temperature on the surface morphology and topography of VA-CNTs. A change in the morphology of the nanotubes between 650 and 700 °C can be observed. Nanotubes grown at a temperature of up to 650 °C show a wave-like morphology, while nanotubes grown at 700 and 750 °C show vertical growth to the substrate.

A qualitative comparison of the wave-like pattern evolution as a function of the deposition temperature shows the morphology transition from a wave-like pattern to a planar pattern when the deposition temperature reaches 700 °C. These results highlight the thermal motion that influences the detachment of the lengthening CNT, allowing us to conclude that the deposition temperature as well as the Ni catalyst–support interactions play a key role in the wave-like pattern formation.

To study and better understand the growth and formation of nanoparticles, SiO_2_ was used as a buffer layer, at different growth temperatures, above and below the previously studied temperature. The surface morphology after the annealing step, for 1 min at 550, 650 and 700 °C, is shown in Fig. 9a, d and g. The Ni nano-sized particles formed at different temperatures are homogeneously distributed over the substrate with a similar high density of nanoparticles. Comparing the morphology, a small difference between diameters can be observed: 19.63, 19.22 and 13.84 nm, corresponding to increasing temperature. The carbon nanotubes grown from these Ni catalyst particles are shown in the SEM images in Fig. 10g–i and m–r, as well as, in the bright-field TEM (BF-TEM) images shown in Fig. 9b, e and h. The VA-CNT arrays, grown at 700 °C, are vertically aligned and planar to the substrate, with a height of 2.2 μm and without any waviness. The range of the external diameters of VA-CNTs is 6.21–16.65 nm, and the average diameter was found to be 9.79 nm.

Nevertheless, at lower temperatures, up to 650 °C (Fig. 10a–o), a wave-like pattern of CNT arrays with aligned and vertical orientation to the substrate can be observed with an average height of CNTs between 0.4 and 3.3 μm, as measured from the cross-sectional SEM images. The amplitude increases and the wavelength of the wave decreases as the temperature increases. These forests of nanotubes completely cover the substrate surface; however, they detach from the surface, where a small forest (0.5 μm in height) of vertically aligned carbon nanotubes grows from the remaining Ni nanoparticles, which we denote as the 2nd layer. As can be seen, the wave-like patterning can be tuned by adjusting the synthesis temperature. A summary of the physical properties of the CNTs at different temperatures is given in Table 3.

**Table 3:** Characterization of the CNT samples produced at different growth temperatures on Si/SiO_2_ substrates. Intensity ratios of the integrated areas of D and G bands and water contact angles (*θ*_c_) of the different CNT arrays obtained

*T* (°C)	Ni nano-sized particle diameter (nm)	Ni nano-sized particle density (cm^−2^)	VA-CNT length (μm)	VA-CNT diameter (nm)	*I*_D_/*I*_G_	*θ*c (°)
450	—	—	0.4	—	—	—
500	—	—	0.7	—	—	—
550	11.63	3.6 × 10^11^	1.5	9.54	1.64	16.01
600	—	—	3.3	—	—	37.20
650	19.22	8.7 × 10^10^	2.3	11.25	1.45	59.24
700	13.84	2.6 × 10^10^	2.2	9.79	1.03	128.16
750	—	—	1.4	—	—	—

**Fig. 11:**
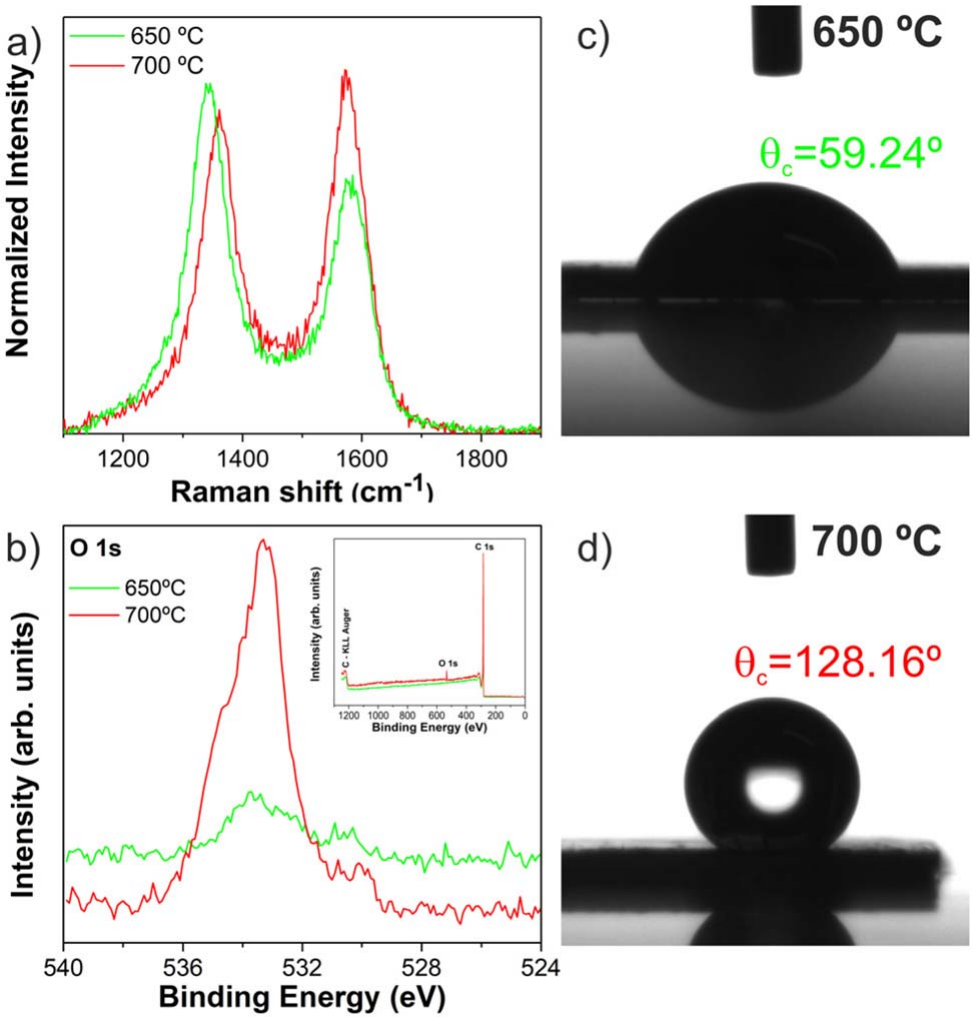
Raman spectra (a) and XPS core-level spectra of O 1s (b); the inset shows the survey spectra of w-VA-CNTs (650 °C) and VA-CNTs (700 °C). Water contact angles of w-VA-CNTs (c) and VA-CNTs (d).

Raman spectroscopy was used to study the degree of crystallinity of carbon nanotubes. As shown in Fig. 11a, the Raman spectrum exhibits two main bands around 1340 cm^−1^ and around 1580 cm^−1^, which represent the D band and G band, respectively, characteristics of multi-walled CNTs.^18^ The intensity ratio of integrated areas of D and G bands (*I*_D_/*I*_G_) is usually applied to evaluate these defects; in other words, lower ratios mean a good degree of crystallinity of the CNTs. The intensity ratios of the different samples are in Table 3. Raman spectra of CNTs produced at 650 and 700 °C are shown in Fig. 11a. In this case, the ratio *I*_D_/*I*_G_ of the VA-CNT arrays grown at 650 and 700 °C was estimated to be around 1.45 and 1.03, respectively, pointing to a higher degree of disorder or defects, when CNTs are produced at lower temperatures. Therefore, these values present a higher degree of disorder or defects compared with CNTs with a good degree of crystallinity.^20^ This result corroborates with the obtained morphologies and internal structure from HRTEM of the CNT arrays, since w-VA-CNTs have a higher degree of structural defects and, in contrast, VA-CNTs have a lower *I*_D_/*I*_G_ value. In Table 3 is presented a summary of characterization of all w-VA-CNTs and VA-CNTs.

### Surface characteristics and adsorption tests

The physical and chemical properties of nanotubes can influence adsorption phenomena. Therefore, specific surface area (SSA) values and the amount of oxygen present on the surface of each forest (w-VA-CNTs and VA-CNTs, grown at 650 and 700 °C) were determined, to evaluate the impact of the two different morphologies. The SSA was calculated based on Peigney *et al.*^21^ and every step is shown in the ESI† and the percentage of oxygen was calculated using XPS measurements. Table 4 summarizes the determined SSA values, O at%, and the % of the removed dyes for the VA-CNTs with and without a wave pattern.

**Table 4:** Determined values of SSA, O at%, % RhB and % MO removed

Sample	SSA (m^2^ g^−1^)	At% (O)	% RhB removed	% MO removed
w-VA-CNTs	180.70	0.62	16.98	3.61
VA-CNTs	191.36	2.32	23.19	0.56

The SSA values are 180.70 and 191.36 m^2^ g^−1^ and they are in accordance with the literature value of 290 ± 170 m^2^ g^−1^.^22^ The VA-CNTs have a higher specific surface area than the w-VA-CNTs. This is due to the slightly different diameters of the nanotubes. Also, the information regarding the chemical and bonding environment of w-VA-CNTs and VA-CNTs was ascertained using XPS (Fig. 11b). The XPS survey spectra (inset image) exhibit two main peaks attributed to O and C elements present in the VA-CNTs.

From the O 1s XPS core-level spectra, the oxygen percentage (O at%) present in the nanotubes was estimated to be 0.62% for the w-VA-CNTs (O 1s peak position, 533.9 eV) and 2.32% on VA-CNTs without a pattern (O 1s peak position, 533.3 eV), exhibiting a significant increase in the relative proportion of the C–O bonds. Interestingly, the oxygen percentage is higher for the VA-CNTs (700 °C) without a pattern, and it seems that O at% behaves differently according to the deposition temperature. The presence of –OH groups on the carbon nanotube surface was also emphasized by measuring the water contact angle (*θ*_c_), depicted in Fig. 11c and d. A *θ*_c_ value of 59.24° was observed for w-VA-CNT forests. As the water contact angle of w-VA-CNTs is less than 90°, the surface is hydrophilic. However, this value changes from hydrophilic to hydrophobic (128.16°) for the VA-CNTs. This contact angle value supports the analysis of the O 1s core-level spectrum conducted in the XPS studies.

**Fig. 12:**
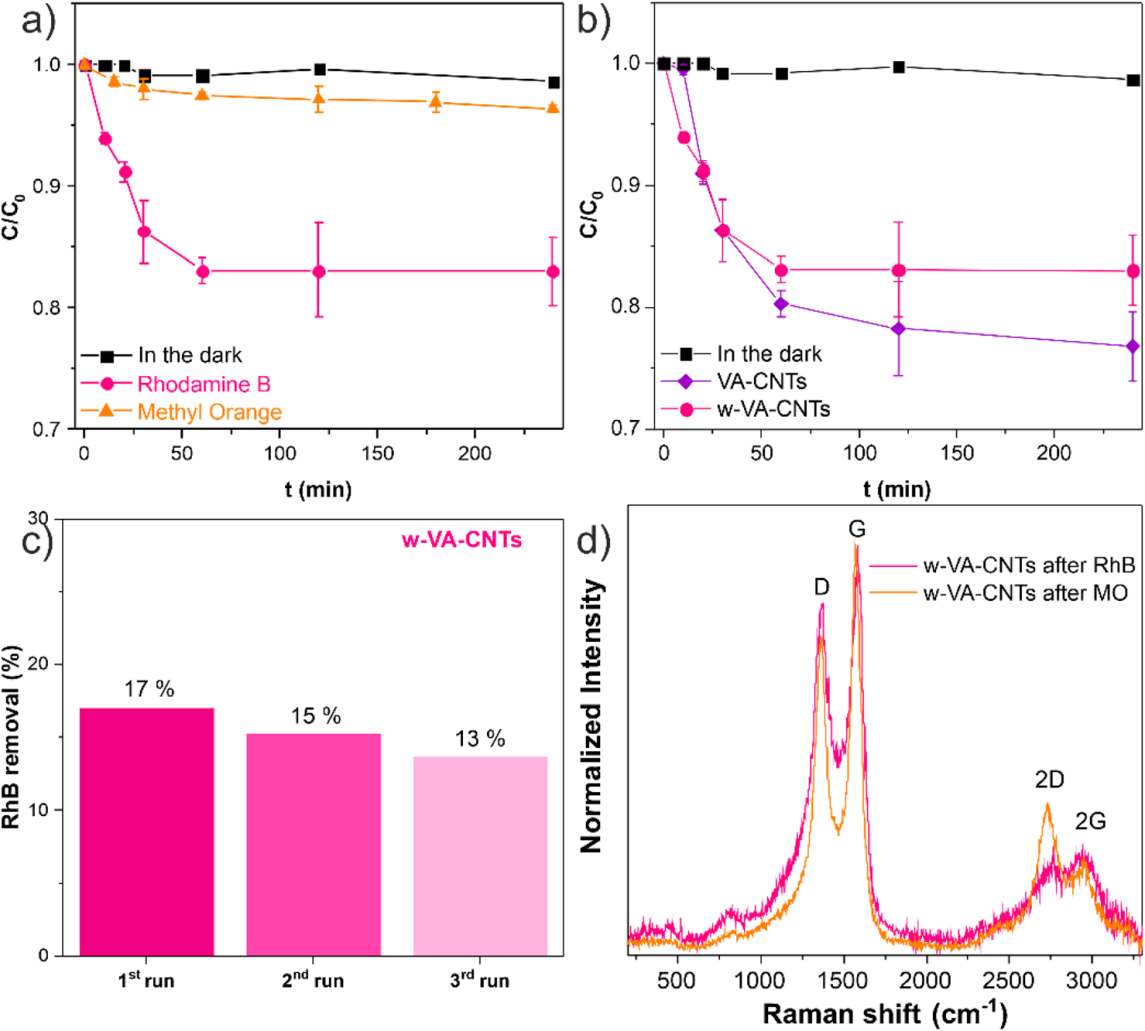
Evolution of the normalized concentration of organic pollutants (*C*/*C*_0_) *vs.* time of adsorption of w-VA-CNTs comparing RhB and MO (a). Evolution of the normalized concentration of RhB (*C*/*C*_0_) *vs.* time of adsorption of w-VA-CNTs (650 °C) *vs.* VA-CNTs (700 °C) (b). RhB degradation percentage of w-VA-CNTs after 3 cycles of recyclability (c). Raman spectra of the w-VA-CNTs after the adsorption tests using RhB and MO (d).

The adsorption tests were performed using two different organic dyes, a cationic dye, Rhodamine B (RhB+) and an anionic dye, methyl orange (MO−). Fig. 12 shows the evolution of both dyes' removal over 240 min of adsorption.

**Fig. 13:**
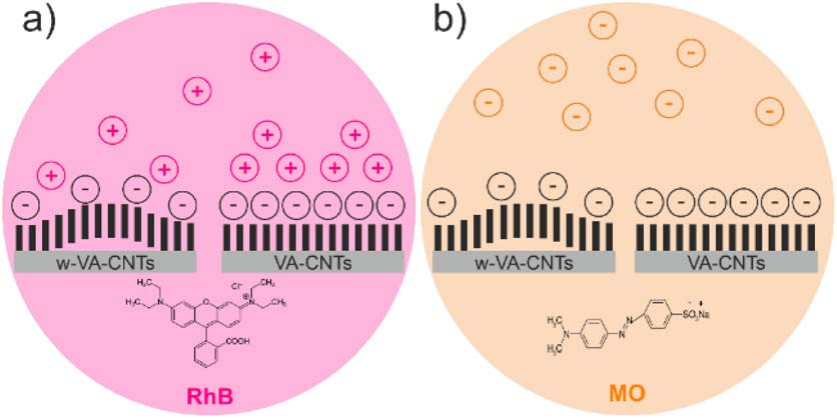
Graphical representation of the spontaneous process during the reaction for w-VA-CNTs and VA-CNTs, when using RhB (a) and MO (b).

Fig. 12a shows that w-VA-CNTs can adsorb around 16.98% of RhB and around 3.61% of MO after 240 min. This result is explained by the negative charges on the nanotube surface and the chemical nature of the dyes. Rhodamine B (RhB) is a cationic dye that contains cationic functional groups; in contrast, methyl orange (MO) is an anionic dye, which means that when in contact with a chemically more negative surface, it is not adsorbed. As would be expected, with an increasing number of surface oxygen groups, as well as increasing specific surface area, from w-VA-CNTs to VA-CNTs, there is an increase in the amount of RhB removed. However, when using MO instead of RhB, the result is completely different because of their chemical nature. Hence, both VA-CNTs are more favourable to adsorb cationic dyes. Fig. 13 shows a schematic representation of the w-VA-CNT and VA-CNT surfaces, dyes, and their interactions.

The w-VA-CNT physical structure, after the adsorption experiments, was analysed by Raman spectroscopy. Fig. 12d shows the two characteristic bands (D- and G-bands) of both samples, implying that the adsorption experiments did not modify the carbon nanotube's physical structure, also supported by XRD measurements (see also Fig. S3†). However, there is a difference in the 2D-band (∼2700 cm^−1^) between the two Raman spectra. The 2D-band or G′-band is a second order Raman peak, with approximately twice the frequency of the D-band.^23^ This finding suggests that the RhB molecules interacted with the nanotubes through electrostatic interactions, since the 2D-band is known to be sensitive to the changes in the electronic structure of the nanotubes.^23^ On the other hand, the interaction of the MO molecules with the nanotubes was significantly low, supported by the minimal adsorption percentage value and the prominent 2D-band. It can be concluded that the nanotubes' intrinsic oxygen generates a negatively charged surface that favours the adsorption of cationic organic dyes in aqueous media, and it has been demonstrated that when oxygen species are present in the nanotubes, they are expected to be negatively charged with higher negative potentials estimated by means of zeta potential measurements.^24^ Finally, the possibility of reusing w-VA-CNTs was assessed by repeating the RhB adsorption process three times. Fig. 12c shows that w-VA-CNTs maintained their adsorption activity during the experiments, suggesting good stability of this material under the reaction conditions used, without a significant loss of adsorption capacity and remained mechanically attached to the support.

## Conclusions

The understanding of the VA-CNT growth mechanism in TCVD is complex and still the subject of ongoing investigations. In fact, improving the understanding of the growth mechanism is particularly complicated due to the variety of experimental systems in terms of the catalyst, support, carbon precursor and growth conditions.

The selection of Ni used as the catalyst was based on the system energy reduction of the TCVD reactions, as demonstrated by the deposition temperature variation ranging from 450 °C to 750 °C. This clearly indicates that the activity of the Ni catalyst starts at 450 °C. The pretreatment step (*i.e.*, annealing in a reductive atmosphere) of the Ni catalyst enhances the growth of the VA-CNTs and they are multiwalled nanotubes. The difference in the morphology of the Ni catalyst is another important result of this study because the size and density of particles largely influence the growth of the VA-CNTs. In addition, the surface after removing the VA-CNTs from the Si substrate implies that the pretreatment treatment leads to a smaller particle size and a larger number of particles than those after the growth step (at 550 and 700 °C). The largest diameter and the lowest density of nanosized Ni catalyst particles were achieved on a sputtered TiO_2_ buffer layer, and the highest VA-CNT length was obtained on it with a planar morphology. These observations might be explained by Ostwald ripening phenomena of the nanosized Ni catalyst on sputtered TiO_2_. The variation in the surface morphology (wave-like and planar) of the VA-CNTs has been correlated with the type of multi-buffer layer used as well as the catalyst–support (*i.e.*, buffer layer) interactions. The Ni catalyst oxidation state plays a key role in the growth of the VA-CNTs. The XPS core-level studies suggest that the degree of the Ni metallic state is more pronounced for the TiO_2_ support, which influences the VA-CNT growth yield.

On the other hand, the intrinsic oxygen concentration was evaluated by XPS core-level analysis, which generates a negative charge favourable for the adsorption of cationic organic dyes in aqueous media. The results also indicated that the intrinsic oxygen concentration of multiwalled CNTs had a greater influence on adsorption than the estimated SSA for w-VA-CNTs and VA-CNTs, implying that the electrostatic interaction may be the main adsorption mechanism in the adsorption process. In addition, the adsorption selectivity of w-VA-CNTs and VA-CNTs in multi-component pollutant systems will be considered as the focus of future studies, which is a very important feature for practical applications towards efficient wastewater decontamination.

## Data availability

The ESI† provides additional details on the experimental section regarding the experimental parameters for the multilayer catalyst preparation and for the several VA-CNT synthesis experiments conducted, as well as calculations of the specific surface areas of the carbon nanotubes.

## Author contributions

Inês E. Oliveira: conceptualization, methodology, investigation, validation, formal analysis, writing – original draft, and writing – review and editing; Ricardo M. Silva: methodology, formal analysis, writing – original draft, and writing – review and editing; Cláudia G. Silva and Rui F. Silva: conceptualization, methodology, validation, supervision, resources, funding acquisition, and writing – review and editing.

## Conflicts of interest

There are no conflicts to declare.

## Supplementary Material

NA-OLF-D4NA00915K-s001
