# Supplementary material for: Modelling VA-CNT surface morphology for pollutant adsorption from aqueous media
Source: Nanoscale Adv. 2025 Jan 22;7(6):1714–26. doi: 10.1039/d4na00915k (PMC11788671; doi:10.1039/d4na00915k)
Supplement: NA-007-D4NA00915K-s001 [file NA-007-D4NA00915K-s001.pdf]

Supplementary Information (SI) for Nanoscale Advances.
This journal is © The Royal Society of Chemistry 2025

# Supplementary information:

# Modelling VA-CNTs surface morphology towards pollutants adsorption from aqueous media

I. E. Oliveira[1,2,3], R. M. Silva[1], C. G. Silva[2,3], R. F. Silva[1]

[1] CICECO – Aveiro Institute of Materials, Department of Materials and Ceramic Engineering, University of Aveiro, 3810-193 Aveiro, Portugal

[2] Laboratory of Separation and Reaction Engineering – Laboratory of Catalysis and Materials (LSRE-LCM), Faculty of Engineering, University of Porto, Rua Dr Roberto Frias s/n, 4200-465 Porto, Portugal.

[3] ALiCE – Associate Laboratory in Chemical Engineering, Faculty of Engineering, University of Porto, Rua Dr Roberto Frias, 4200-465 Porto, Portugal

**Corresponding author:**

## Experimental details

Table S1 and Table S2 summarize the experimental parameters corresponding to the multilayer catalyst preparation and the experimental parameters corresponding to the several VA-CNTs synthesis carried out, where different values of temperatures were tested, respectively.

Table S1. PVD deposition parameters for the different types of thin films.

| Parameters | $Al_2O_3$ | $TiO_2$ | $Cr_2O_3$ | Ni |
|---|---|---|---|---|
| **Target** | Al | Ti | Cr | Ni |
| **Base Pressure (mbar)** | 3.5x10$^{-5}$ | 3.5x10$^{-5}$ | 3.5x10$^{-5}$ | 3.5x10$^{-5}$ |
| **Working pressure (mbar)** | 5.2x10$^{-4}$ | 5.2x10$^{-4}$ | 4.6x10$^{-4}$ | 4.4x10$^{-4}$ |
| **Current (A)** | 0.02 | 0.06 | 0.08 | 0.02 |
| **Power input (W)** | 20 | 20 | 20 | 20 |
| **Flux of Ar (sccm)** | 10 | 10 | 10 | 10 |
| **Flux of $O_2$ (sccm)** | 4 | 4 | 4 | - |
| **Deposition time (min)** | 360 | 360 | 70 | 6, **12**, 19, 30, 60, 120, 240 |

Table S2. Experimental conditions of VA-CNTs synthesis by TCVD.

| Parameters | Pre-treatment | Growth |
|---|---|---|
| **Temperature (°C)** | 450, 500, 550, 600, **650**, 700, 725, 750 | |
| **Flux of Ar (sccm)** | 200 | **400**, 450, 500 |
| **Flux of $H_2$ (sccm)** | 500 | 0, 50, **100** |
| **Flux of $C_2H_2$ (sccm)** | - | 10 |
| **Deposition time (min)** | 0.5, **1** | **15** |

## Specific surface area of CNTs

The calculations for obtaining the specific surface area of multi-walled carbon nanotubes ($SSA_{MWCNTs}$) are based on the following premises: (i) all CNTs are closed, and thus only the external surface of each CNT is taken into account, (ii) the length of the C–C bonds ($d_{C\text{-}C}$) in the curved graphene sheets is the same as in the planar sheet (Figure S1d), (iii) the MWCNTs are composed of concentric graphene sheets (Figure S1c) and with a wall-wall distance ($d_{W\text{-}W}$) (Figure S1b and c), (iv) the aspect ratio of CNTs is sufficiently high to neglect the area of the tip surfaces in comparison to the area of the cylindrical surfaces.

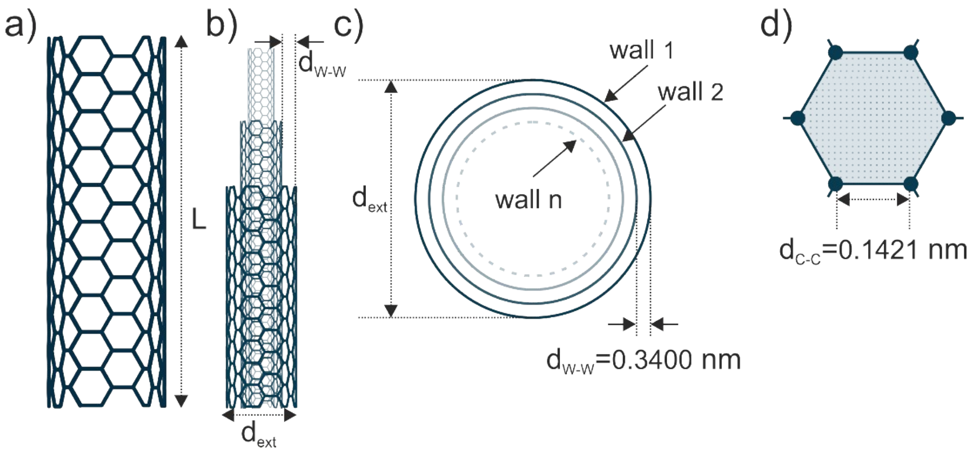


Figure S1. Schematic representation of SWCNTs (a) MWCNTs (b) MWCNTs made up of concentric graphene walls (c) and the arrangement of carbon atoms within the graphene layer (d), adapted from [1].

The following Equations are based on Peigney *et al.* [1], where $S_{MWCNT}$ is the surface area and $W_{MWCNT}$ is the weight of MWCNT. It is taken into consideration that MWCNT with

an external diameter $d_{ext}$, a length $L$ and a $n$ number of walls (Figure S1a, b and c). In MWCNTs, $n \geq 2$.

$$S_{MWCNT} = \pi L d_{ext} \ (m^2)$$ Equation 1

where L corresponds to the length.

$$W_{MWCNT} = {}^{1}\!/_{SSA(graphene)} \times \pi L \left( n d_{ext} - 2 d_{w-w} \sum_{i=1}^{n-1} i \right) \ (g)$$ Equation 2

Combining Equation 1 and 2 gives the specific surface area of MWCNT ($SSA_{MWCNT}$):

$$SSA_{MWCNT} = {}^{S_{MWCNTs}}\!/_{W_{MWCNT}} = {}^{1315 d_{ext}}\!\Big/_{n d_{ext} - 2 d_{w-w} \left[ \sum_{i=1}^{n-1} i \right]} \ (m^2 g^{-1})$$ Equation 3

Figure S2 are bright-field and high-resolution TEM images of the studied of the two types of the studied nanotubes with the all the parameters used for the calculations.

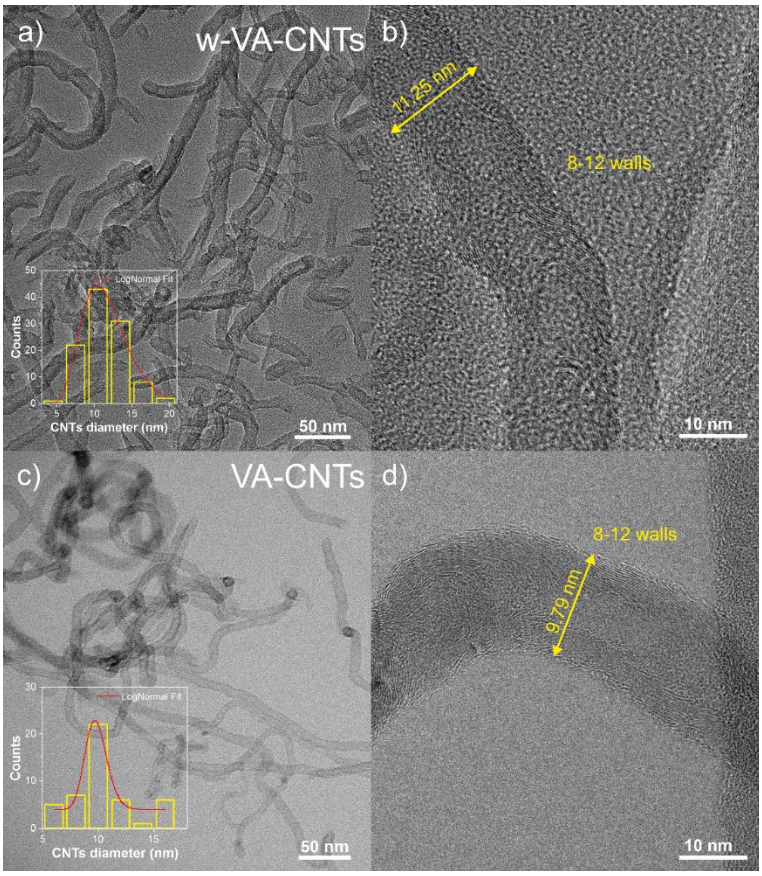


Figure S2. Overview TEM and HRTEM of w-VA-CNTs (a, b) and VA-CNTs (c, d). The inset contains the CNTs diameter distribution.

The Table S3 summarizes the parameters used for the calculations with the information from HRTEM images.

Table S3. Parameters used for the determination of specific surface area.

| Parameters | | w-VA-CNTs | VA-CNTs |
|---|---|---|---|
| $L$ | µm | 2.3 | 2.2 |
| | m | $2.3\times10^{-6}$ | $2.2\times10^{-6}$ |
| $d_{ext}$ | nm | 11.25 | 9.79 |
| | m | $1.125\times10^{-8}$ | $9.790\times10^{-9}$ |
| $n$ | | 10 | 10 |
| $d_{C\text{-}C}$ | nm | 0.1421 | |
| | m | $1.421\times10^{-10}$ | |
| $d_{W\text{-}W}$ | nm | 0.3400 | |
| | m | $3.400\times10^{-10}$ | |
| **SSA** | $m^2g^{-1}$ | 180.70 | 191.36 |

## Results and discussion

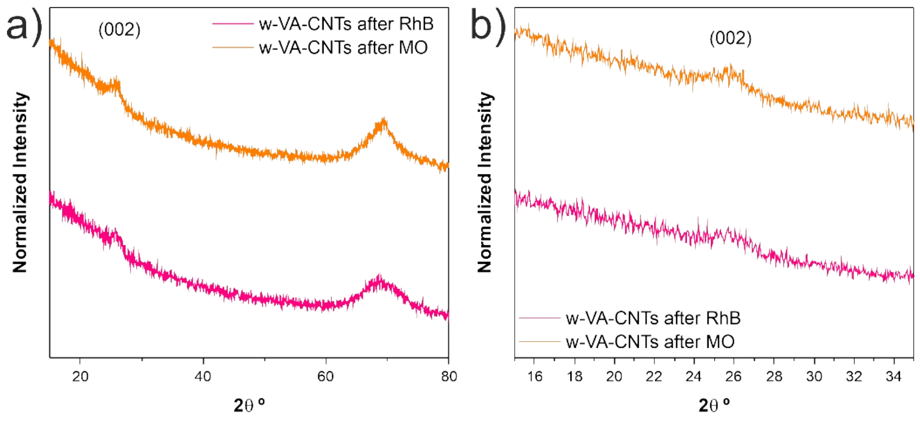


Figure S3. (a) XRD diffraction patterns of w-VA-CNTs after RhB and MO adsorptions tests. (b) XRD diffraction pattern in the 15 - 35 ° 2θ range, highlighting the (002) plane without any noticeable changes. The observable prominent peak is related to the Si/$SiO_2$ substrate.

## References

[1] Peigney A, Laurent C, Flahaut E, Bacsa RRR, Rousset A. Specific surface area of carbon nanotubes and bundles of carbon nanotubes. Carbon. 2001; 39(4):507-514. doi:10.1016/S0008-6223(00)00155-X
